# The evolution of our understanding of human development over the last 10 years

**DOI:** 10.1038/s41467-021-24793-3

**Published:** 2021-07-29

**Authors:** Ali H. Brivanlou, Norbert Gleicher

**Affiliations:** 1 Stem Cell Biology and Molecular Embryology Laboratory, The Rockefeller University, New York, NY USA; 2 The Center for Human Reproduction, New York, NY USA; 3 The Foundation for Reproductive Medicine, New York, NY USA; 4 Department of Obstetrics and Gynecology, Medical University of Vienna, Vienna, Austria

**Keywords:** Developmental biology, Embryogenesis

## Abstract

As it fulfills an irresistible need to understand our own origins, research on human development occupies a unique niche in scientific and medical research. In this Comment, we explore the progress in our understanding of human development over the past 10 years. The focus is on basic research, clinical applications, and ethical considerations.

## What basic research has taught us about human development

Over the last decade, progress in understanding our own development was mostly driven by the emergence and combination of remarkable new technologies. New molecular biology tools such as single-cell RNA-sequencing (sc-RNA-seq) unveiled the earliest genetic signature of the three cell lineages of the human blastocyst and allowed for the discovery of human-specific signatures^1–3^. CRISPR/Cas9 genome editing has offered further access to in vitro functional studies in human blastocysts^4^. However, as we discuss below, an ethical line was crossed when a group claimed that genetically modified human embryos had been transferred, leading to births^5^ when neither public opinion nor a consensus within the scientific community had been reached regarding whether crossing the germline in in vitro fertilization (IVF) was safe and ethically acceptable.

On the embryology side, the development of an in vitro attachment platform for human blastocysts offered a first glance into post-implantation events up to 12 days^1,3,5,6^. This paved the way for several important discoveries, including the observation that the human embryo can self-organize to generate embryonic and extraembryonic germ layers, yolk sac, and amniotic cavities in the absence of maternal influences^5,6^; and the presence of a transient embryonic tissue of trophectodermal lineage, adjacent to the yolk sac, therefore named, *yolk-sac trophectoderm* (*ysTE*)^5^. The presence of these seemingly human-specific populations was independently confirmed by sc-RNA-seq^1^.

The marriage of stem cell biology with bioengineering gave birth to the field of synthetic embryology^7–13^. This technology uses human embryonic stem cells (hESCs) cultured on geometrically confined micropatterned substrates to generate 2D in vitro models of human conceptuses, such as models of the gastrula (*gastruloids*)^7–13^, or parts of the embryo, such as *cerebroids* and *neuruloids*^14^. Thousands of nearly identical self-organizing human embryonic structures allow for standardization and reproducibility, which cannot be achieved in standard organoid structures^15^. Cells within these structures can be tracked and quantified in real time with sub-cellular resolution, using sophisticated quantification code, including artificial intelligence^14^.

Human *gastruloids* induce formation of the primitive streak and have enabled the deciphering of the molecular network underlying gastrulation—the most crucial moment of our lives^7–13^. 3D models of human epiblasts can spontaneously break axial symmetry, thus providing an assay for the elucidation of molecular events underlying the emergence of antero–posterior polarity^11,16^. A highly homogenous population of self-organizing 3D models of amniotic ectoderm-like cells can be obtained by combining microfluidic and microculture approaches^17^.

Finally, the development of interspecies chimeras provided the most stringent in vivo validation of human embryo models^9,10,18^. Unimaginable in human models, inter-species chimeras have become the next best choice to test whether hESC behavior in self-organizing *gastruloids*, as observed on microchips, would also occur in an embryonic environment^10,18,19^. Human/bird chimeras generated from transplanting human gastruloids into early chick embryos in ovo unexpectedly proved more efficient than previous methods^9,19^. They allowed for the observation of an entire self-organizing embryonic axis in bird eggs^9^. As birds are closer to dinosaurs than to humans, this high rate of success with these chimeras further suggested that these early patterning events must be highly conserved.

## Translational clinical applications that arose from basic research

The past 10 years bore witness to significant clinical progress in reproductive medicine, often translated from basic research. Successful human uterus transplantation and the subsequent birth of healthy offspring was, for example, only achieved after years of meticulous laboratory work in animals^10^. Significant improvements in cryopreservation technology for human eggs and ovarian tissue were also preceded by research in model systems^10,20^. Practical clinical applications have been developed for women in need of cancer treatment that are toxic to ovaries. In these cases, oocytes and/or ovarian tissue can be cryopreserved for later use in fertility treatments once the patient is cured of her cancer^21^. This ever-evolving technology has already proven to result in live births, and has also become an integral part of routine infertility treatments with IVF, giving rise to the brand-new concept of fertility extension through egg-freezing.

Diagnostic technologies to assess retrieved eggs and preimplantation-stage embryos in the IVF process have been disappointing. For example, tracking extended embryo culture to blastocyst-stage with time-lapse imaging failed to improve embryo selection^22^. That chromosomal-abnormal embryos increase with maternal (but not paternal) age has been interpreted to mean that chromosomal abnormalities were a principal cause for lower implantation chances and higher miscarriage risks among older women. This assumption led to the rapidly growing utilization of chromosomal testing of human embryos prior to embryo transfer in a procedure recently renamed preimplantation genetic testing for aneuploidy (PGT-A)^23^. The hypothesis behind PGT-A is to exclude chromosomal-abnormal embryos from the transfer, thereby improving implantation potentials of remaining euploid embryos.

Here too, clinical evidence was unable to confirm the hypothesis^24^. Moreover, basic research demonstrated a self-correction mechanism in mouse^25^ and human embryos^26–29^ that arose during embryogenesis that was cell lineage-specific to the embryonic cell lineage. In contrast, PGT-A biopsies are obtained from the extraembryonic-derived trophectoderm, rendering any diagnostic procedure at the blastocyst stage ineffective. In addition, mathematical modeling demonstrated that results from a single trophectoderm biopsy could not be extrapolated to the whole embryo^30^. Transfer of PGT-A “chromosomal-abnormal diagnosed embryos” has resulted in the births of over 400 chromosomal-normal offspring^20,21^.

In recent years, increasing attention has also been given to the quickly evolving understanding of how interdependent lifestyle and human fertility are^31–33^, including the influence of diet on the microbiome, as in many other areas of medicine.

## The ethical significance of understanding human development

Whether in clinical medicine or in the research laboratory, human embryology has remained an ethical minefield, strongly influenced by socio-political and religious considerations. At the core of the controversy resides the special moral value of the human embryo, a subject that has come to the forefront again with the ascent of human embryonic stem cell research^34^. There is, however, little consensus as to how to answer a previously raised question: “*what is an embryo*?”^35^. The term pre-embryo, first introduced in 1986, was defined as the interval up to the appearance of the primitive streak, which marks biological individuation at ~14 days post-fertilization. This definition designated the period beyond 14 days as the time when a pre-embryo attains special moral status^36,37^. Paradoxically, the term pre-embryo has been replaced by the indiscriminate use of the term embryo, whether at preimplantation cleavage or blastocyst-stages or post-implantation before day 14. It was suggested that the distinction was important for ethical, moral, and biological relevance. The principal reason is simple: Until a pre-embryo becomes an embryo, there is no way of knowing whether implantation has taken place, whether a pregnancy is developing, whether there is a single pregnancy or twinning, or whether fertilization ended up in a benign (hydatidiform mole) or even in a malignant tumor (choriocarcinoma)^35^. Assigning advanced moral value to embryos at those early stages is, therefore, difficult to defend.

The past 10 years have witnessed innumerous ethical debates related to this subject, each with its own social, historical, and religious justifications, reflecting cultural diversities in human populations. Most are triggered by scientific breakthroughs. We summarize here the major ethical challenges preoccupying reproductive research and clinical practice.

We have already briefly referred to CRISPR/Cas9 genome editing. While the use of sc-RNA-seq to identify the molecular blueprint of human development has not elicited significant controversy, CRISPR/Cas9 genome editing of human embryos has been a topic of intense discussions and is currently permissible only in vitro^38^. An alleged attempt in China of implanting human genome-edited embryos into the uterus supposedly led to two births (one a twin birth). Though widely discussed in the media, neither attempt was published in the medical literature, and therefore cannot be verified^5,38^.

The ethical debates surrounding the 14-day rule, quiescent since the early IVF days, experienced a rebirth that was prompted by in vitro human embryo attachment studies and the emergence of synthetic human embryos. Within this context, we note that self-organizing embryo models are nothing more than cells in culture and are certainly not embryos. Regardless of scientific merits, in the U.S., the National Institutes of Health (NIH) currently prohibits the use of public funds for the study of synthetic embryos “for ethical reasons”. After being under an NIH moratorium for more than a year, research on chimeras is now, however, again permitted, though human/non-human primate chimeras remain prohibited.

These ongoing ethical debates mostly also mirror those surrounding the lack of U.S. federal funding for clinical IVF and related research, as well as hESCs-derived model embryos. In this context, the American Society for Reproductive Medicine (ASRM)’s Ethics in Embryo Research Task Force recently made an important statement: “*Scientific research using human embryos advances human health and provides vital insights into reproduction and disease*”^39^.

Provided certain guidelines and safeguards are followed, research with already existing embryos or embryos specifically produced for research should be ethically acceptable as a means of obtaining new knowledge that may benefit human health. ASRM also pointed out that scientists and society must understand which research questions necessitate the use of human embryos.

It is gratifying to acknowledge the history and vitality of ongoing debates, especially since they increasingly mimic decision-making processes in the medical field. These debates are meant to be based on cost-benefit and/or risk-benefit assessments. These debates will, unquestionably, continue and, indeed, considering that every intervention has consequences, must be decided based on careful considerations, including all relevant stakeholders and all parts of society.
